# Supplementary material for: Development of a Scale to Measure Infant Eating Behaviour Worldwide
Source: Nutrients. 2021 Jul 22;13(8):2495. doi: 10.3390/nu13082495 (PMC8398650; doi:10.3390/nu13082495)

Supplementary tables

Table S 1: Timetable and sequencing of development of eating behaviour scales for International Complementary Feeding Evaluation tool (ICFET)

	**Early work**	
1999	Initial eating behaviour (EB) questions collated and applied to GMS cohort[9]	
2009	Second analysis of GMS EB data to form appetite scale, broadly unsuccessful[26]	
2014-16	First draft of $ICFET_1$ including GMS EB phrases developed and tested in Kenya and Ghana[18]	
	**Video study with parents**	**Survey work with parents and children**
2016-17	GMS EB phrases supplemented with selected CEBQ items to form 1st prototype; video methodology developed and piloted in UK (English and Urdu) and EB phrases refined to form 2nd prototype	$ICFET_2$ developed including 2nd prototype EB phrases and translated into Urdu
2018	Video study in UK using 2nd EB prototype phrases (English and Urdu) and Hong Kong (Cantonese)	$ICFET_2$ tested in Pakistan
2019	Video study in Cyprus and Indonesia	$ICFET_2$ tested in UK, translated into Swahili and Spanish and tested in Kenya and Guatemala<br><br>Behaviour questions from $ICFET_2$ tested in Cyprus and Indonesia

Table S 2: Phrases used at different stages and their sources

*GMS phrases §CEBQ

Linguistic study pilot

Avidity	Avoidance
Enjoys eating §<br>Loves food*<br>Finishes his/her meal quickly §<br>Is interested in food §<br>Is always asking for food §<br>Enjoys a wide variety of foods §<br>Enjoys eating §<br>Looks forward to mealtimes §	Turns away *<br>Pushes food away*<br>Cries/ screams*<br>Holds food in mouth*<br>Spits out food*<br>Eats slowly §<br>Leaves food on his/her plate at the end of a meal §

Linguistic study definitive

Avidity	Avoidance
Enjoys eating §<br>Loves food* (translated as likes food a lot in most languages)<br>Is interested in food §<br>Is always asking for food §<br>Enjoys a wide variety of food §<br>Eats quickly (modified §)<br>Is willing to try new foods (modified §)<br>Finishes his/ her meal (modified §)	Turns head away (modified*)<br>Pushes food away*<br>Cries/ screams*<br>Holds food in mouth for too long (modified*)<br>Spits out food*<br>Meals often last more than 30 minutes<br>Meals sometimes last more than an hour

Final included phrases

Avidity	Avoidance
Enjoys eating §<br>Likes food a lot (modified §)<br>Is interested in food §<br>Enjoys a wide variety of food §<br>Eats quickly (modified §)<br>Finishes his/ her meal (modified §)	Turns head away (modified*)<br>Pushes food away*<br>Cries/ screams*<br>Spits out food*<br>Meals sometimes last more than an hour

Table S 3: Details of sampling in each setting

Study 1	Location	Participants	Recruited via	Ethics	Study delivery	Collected by	Measurements
UK		Adults who had ever parented a child aged more than 6 months	Toddler activity groups, Muslim ladies' groups, snowball and convenience sampling	UoG[1] 200160042	Interview	Students	None
Hong Kong		Adults who had ever parented a child aged more than 6 months	Mother and toddler groups, elderly social clubs and community centres	UoG[1] 200170117	Interview	Student	None
Cyprus		Parents of a child currently aged 6-24 months	Nurseries, social media advertisement and by snowball and convenience sampling	UoG[1] 200180123	Interview	Students	None
Indonesia		Parents of a child currently aged 6-24 months	Community groups & community-based clinics	UoG[1] 200180123	Interview	Student	None
**Study 2**							
UK		Parents and healthy child currently aged 6-24 months	Toddler activity groups	UoG[1] 200180033	Self completion	Students	weight, length
Pakistan		Parents and healthy or undernourished child currently aged 6-24 months	Child health clinics	UoG[1] 200170181	Interview	Student	weight, length, MUAC
Kenya		Parents and healthy or undernourished child currently aged 6-24 months	Child health and nutrition clinics	Kenya[2] P576-2018	Interview	Research assistants	weight, length, MUAC
Guatemala		Parents and healthy or undernourished child currently aged 6-24 months	Community programmes	UoG 200180131; Guatemala[3] CE/FM-UFM 30-19	Interview	Research assistants	weight, length, MUAC
Cyprus		Parents and healthy child currently aged 6-24 months	Nurseries, social media advertisement and by snowball and convenience sampling	UoG 200180123	Self completion	Students	None
Indonesia		Parents of a healthy child currently aged 6-24 months	Community groups & community-based clinics	UoG 200180123	Interview	Student	None

[1]University of Glasgow College of MVLS research ethics committee [2]AMREF ethics and scientific review committee
[3]Comité de ética Independiente Facultad de Medicina Universidad Francisco Marroquín / Hospital Universitario Esperanza

Table S 4: Descriptions of the standard videos used to display different eating behaviours in Study 1

Video	Description
**1: Very Enthusiastic**	Pre-school child sitting at a table with her sister. There is a plate of pasta with cheese in front of her. She eats with her hands, eating very quickly, constantly placing food in her mouth.
**2: Enthusiastic**	Boy aged 15 months is sitting in a highchair with a plate of mashed potato, peas, carrots and stewed meat. He is being fed with cutlery and eats steadily, although not particularly quickly. He asks for food, shows signs of trying to feed himself and shows interest in his meal, picking up food when it falls off the spoon.
**3: Moderate Food refusal**	Three-year-old boy sitting at the table with his mother and a plate of pasta. He is being fed with cutlery but is shown to be distracted and shows little to no interest in his meal. He barely eats in the video.
**4: Severe Food refusal**	A school age boy, but with significant learning disability, at the table with his mother and a plate with plain yogurt. His mother is attempting to feed him with a spoon, but he avoids eating throughout the video, pushing food away when he is being fed.

Table S 5: Study two: numbers in each group, age and weights (when measured) for healthy and underweight children

				**Normal weight**				**Underweight**			
		Age, months				Weight z				Weight z	
	N	Mean	SD	Number	%	Mean	SD	Number	%	Mean	SD
Kenya	157	12.5	(4.0)	107	68%	-0.59	(0.93)	50	32%	-2.81	(0.61)
Pakistan	108	15.7	(5.4)	67	62%	-0.17	(1.16)	41	38%	-3.37	(0.98)
Guatemala	125	15.3	(5.6)	80	64%	-0.96	(0.85)	45	36%	-2.75	(0.59)
UK	107	13.3	(5.5)	85	97%	0.40	(1.03)	3	3%	-2.37	(0.23)
**Total with weights**	**478**	**14.1**	**(5.3)**	**339**	**71%**	**-0.35**	**(1.10)**	**139**	**29%**	**-2.94**	**(0.78)**
Cyprus	26	17.0	(6.3)								
Indonesia	50	13.8	(6.0)								
**Total with ICFET data**	**573**	**14.2**	**(5.4)**								

Supplementary figure: Study 1: Percentage of participants showing full agreement, some uncertainty, or disagreement with each phrase across the four videos

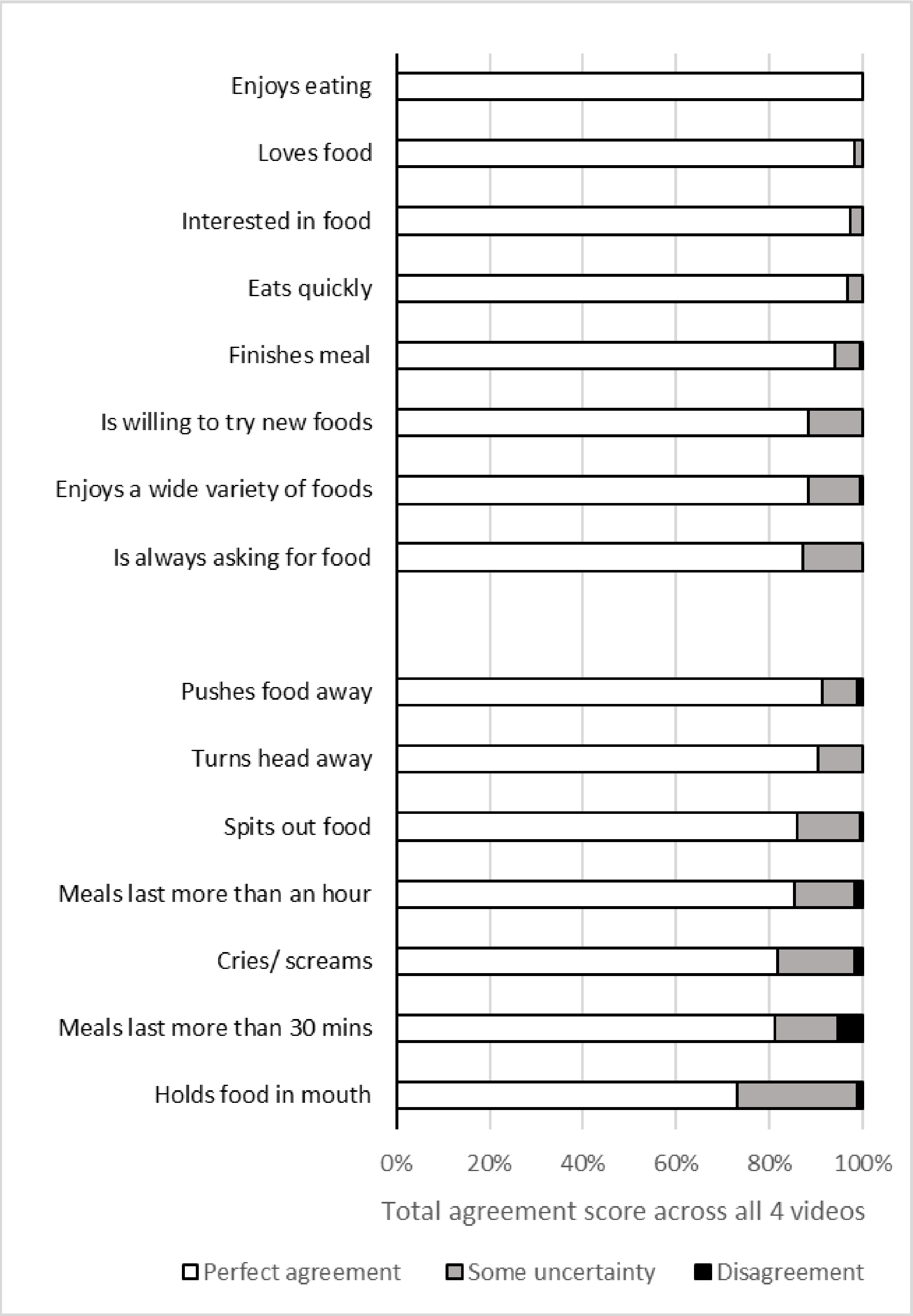

Supplement: Supplementary file 1 [file nutrients-13-02495-s001.zip › nutrients-1284249-supplementary.pdf]
